# Relationship between Khat chewing and upper digestive tract cancers among male patients in Hargeisa: case control study

**DOI:** 10.3332/ecancer.2025.1880

**Published:** 2025-03-25

**Authors:** Abdiwahab M Ali, Mirriam N Mutuku, Abdiwahab Hashi, Omar M Muhumed

**Affiliations:** 1 Amoud University, School of Postgraduate Studies & Research, Department of Public Health, Hargeisa, Somaliland; 2 Amoud University, School of Postgraduate Studies & Research, Hargeisa, Somaliland

**Keywords:** Khat chewing, upper digestive tract cancers (UDT), risk factors, Somaliland

## Abstract

Khat chewing is a common cultural practice in countries bordering the Red Sea and the east coast of Africa. Despite some indications in the literature, its association with upper digestive tract (UDT) cancers is under-researched. This study investigated the relationship between khat chewing and UDT cancers among male patients in Hargeisa using a case-control design. A total of 97 respondents were included, 36 cases and 61 controls from the only two cancer clinics in Somaliland, Nageeye Cancer Clinic and Needle Hospital. The study used non-probability purposive sampling, data collection was conducted using a structured questionnaire, and data entry and cleaning were performed using SPSS version 22. Analysis was carried out using Stata MP 16. The findings revealed an association between duration, frequency and amount of khat chewing and the risk of developing UDT cancers. Specifically, individuals who chewed khat for more than 20 years had a 7.05 times higher risk (*p* < 0.05), those who chewed daily had a 6.89 times higher risk (*p* < 0.05), and heavy chewers (>600 g) had a 6.60 times higher risk (*p* < 0.05) of developing UDT cancers. The associations found in this study between khat chewing and UDT cancers suggest an urgent need for public health strategies, including education and policy reforms, to address and reduce the health risks posed by khat chewing in Somaliland. The study also highlighted the importance of community education and awareness programs to mitigate the adverse health effects of khat.

## Background

Khat (*Catha edulis*), is a stimulant plant native to regions along the Red Sea and East Africa, has been traditionally chewed for centuries in various cultures under different names such as chat, qat and miraa [8]. Despite its widespread use primarily in Arabic, Middle Eastern and African communities, the potential health risks associated with khat consumption have gathered increasing attention, particularly concerning its role in upper digestive tract (UDT) cancers [7].

UDT cancers include malignancies affecting organs like the oral cavity, esophagus and stomach, significant contributors to global cancer incidence and mortality, especially prevalent in low- and middle-income countries [14]. Esophageal cancer (EC), in particular, ranks prominently among these cancers, with Eastern Africa identified as a hotspot region for its high prevalence and mortality rates [6]. Despite this geographic variance, the specific mechanisms linking khat consumption to these cancers remain under-researched.

Research indicates that both the duration and frequency of khat chewing play significant roles in cancer development. For instance, studies in Saudi Arabia and Yemen have associated long-term khat chewing with oral squamous cell carcinoma (OSCC) and EC, respectively [1, 4]. Additionally, a study in Djibouti found a significant association between khat consumption and premalignant oral lesions [5]. Moreover, the amount of khat consumed in each session has been linked to increased risks of dental health problems and potentially higher susceptibility to UDT cancers [16]. A study by Lukandu *et al* [12] pointed out that there was an association between khat use and oral lesions such as hyperkeratosis and oral cancer. A study conducted in Ethiopia by Dessalegn *et al* [9] concluded that tobacco smoking and khat chewing were positive predictors of EC. These findings point out the importance of understanding the health risks associated with khat chewing, particularly in contexts where it is a common cultural practice.

This study aims to address this gap by looking into the relationship between khat chewing and the development of UDT cancers, focusing specifically on oral cavity and ECs. The objectives include evaluating the duration, frequency and amount of khat chewed as potential risk factors for these cancers among male patients in Hargeisa, Somaliland.

## Methods

This study employed a case-control design to investigate the association between khat chewing and UDT cancers among male patients in Hargeisa, Somaliland. Cases were individuals diagnosed with UDT cancers, while controls were selected without such diagnoses. The study included male participants from Hargeisa, Somaliland, who attended Nageeye Cancer Clinic or Needle Hospital. Participants with incomplete data, unclear diagnoses or those unwilling to participate were excluded. The study was designed for 101 participants, comprising 34 cases and 67 controls, calculated to achieve sufficient statistical power with a 2:1 ratio of controls to cases. Using purposive sampling, 110 respondents were selected during data collection to account for potential errors such as incomplete responses. After data cleaning and excluding invalid responses, the final sample size included 97 participants, consisting of 36 cases and 61 controls. Data were collected through structured questionnaires administered via interviews and medical record reviews. The questionnaires covered demographics, khat chewing habits and other relevant behaviors. Medical records provided additional diagnostic and historical data. Ethical clearance was obtained from Amoud University and the participating hospitals. Informed consent was obtained from all participants or their legal representatives. Confidentiality and privacy of participants were strictly maintained throughout the study.

SPSS was used for data entry and cleaning. For data analysis, Stata/MP version 16.0 was employed, including descriptive statistics, bivariate analysis and multivariate logistic regression to assess the association between khat chewing and UDT cancers. Quality control measures included pilot-testing and validation of research instruments to ensure high validity (Content Validity Index of 1.00) and reliability (test-retest reliability coefficient of 0.97). These measures were crucial in maintaining the integrity and accuracy of the study’s findings. Additionally, the study focused only on male participants to ensure validity and reduce social desirability bias. Khat chewing among women is considered taboo and unethical, making it difficult for women to disclose their khat-chewing habits.

## Results

**Figure 1.**
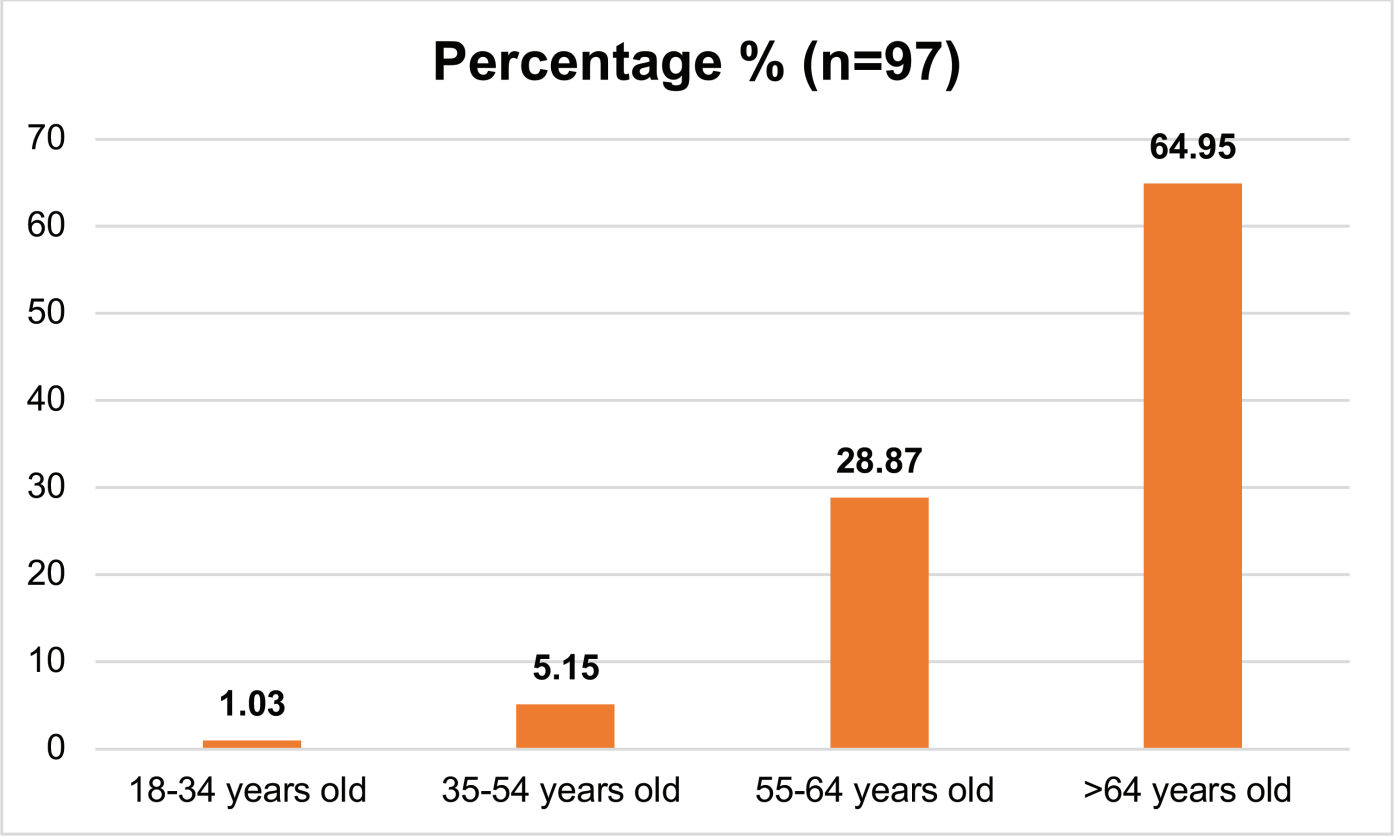
Age of the study respondents.

**Figure 2.**
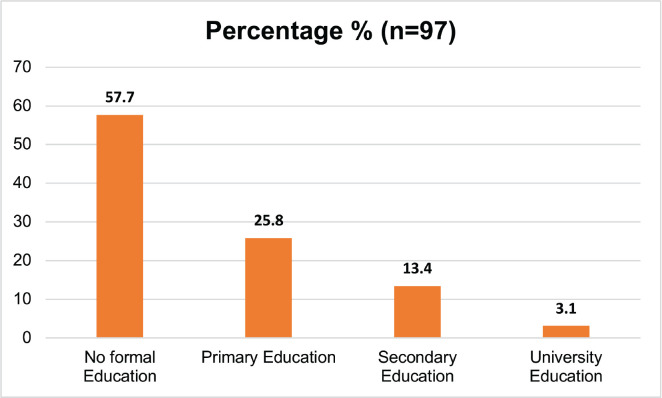
Level of education of the study respondents.

**Figure 3.**
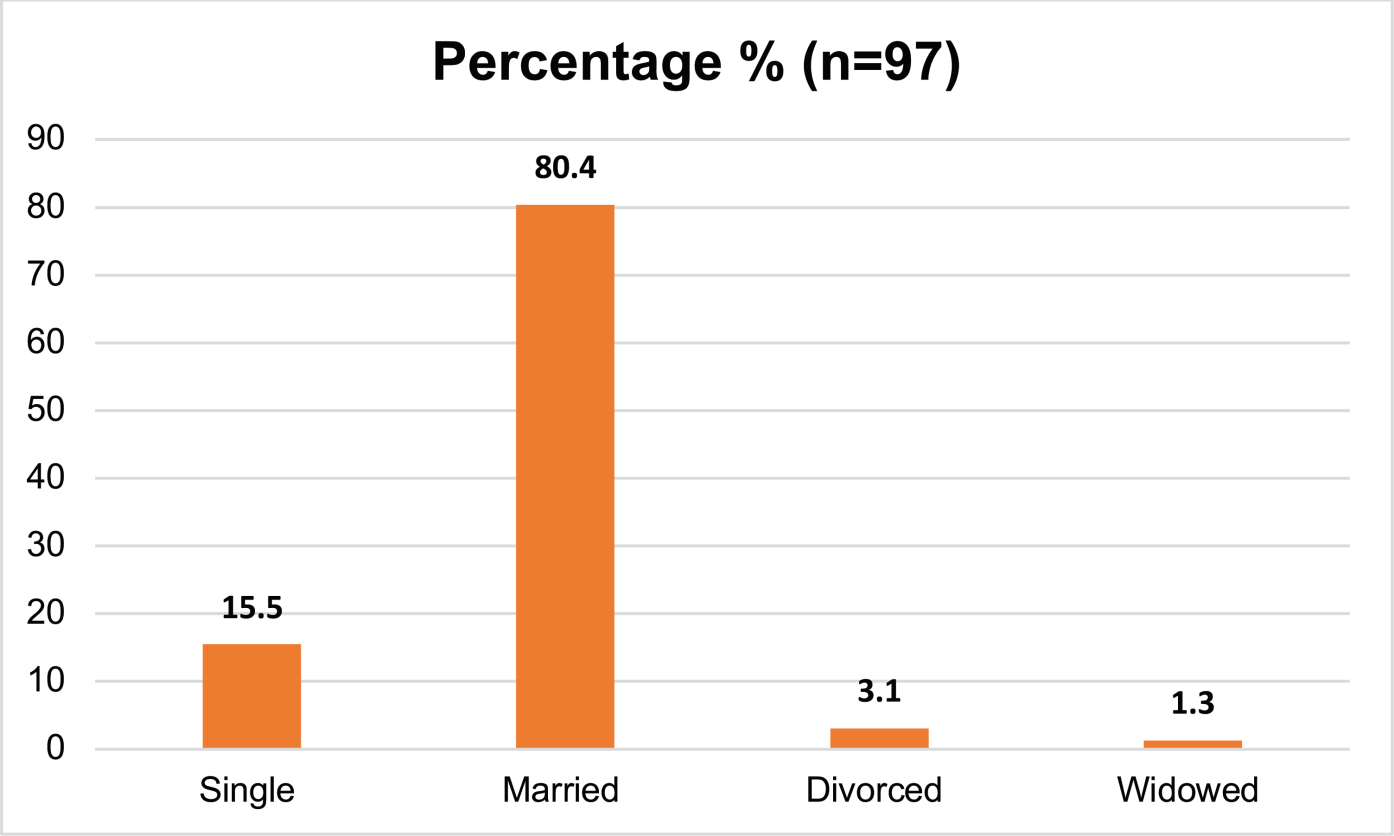
Marital status of the study respondents.

Table 1 describes the demographic and behavioral characteristics of the study participants. 97 respondents were categorised into cases (those with UDT cancer) and controls (those without). Controls made up 62.89% of the group, while cases comprised 37.11%. The participants’ ages varied, with the majority (64.95%) being over 64 years old (Figure 1). Education levels were mostly low, with 57.73% having no formal education (Figure 2). Marital status showed a predominance of married individuals (80.41%) (Figure 3).

**Figure 4.**
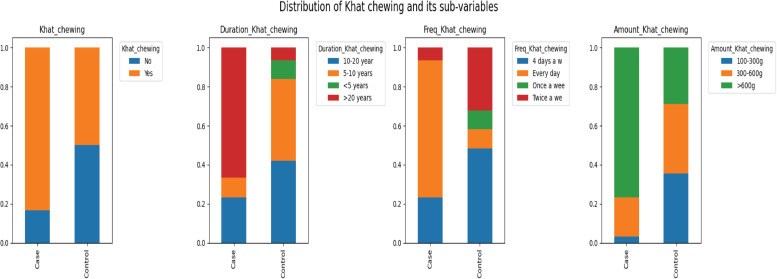
Distribution of Khat chewing and its sub-variables.

A slightly higher percentage of respondents attended Nageeye Cancer Clinic (57.29%) compared to Needle Hospital (42.71%). Among the cases, 63.89% had EC and 36.11% had oral cancer. A significant majority, 63.9%, of the respondents are khat chewers, with a notable portion, 36%, chewing for more than 20 years (Figure 4). The frequency and amount of khat chewing also show concerning trends, with 40.32% chewing every day and 53.23% consuming more than 600 g per session, indicating a high level of exposure among regular users.

**Figure 5.**
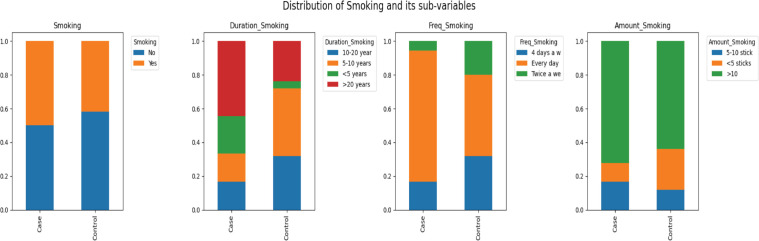
Distribution of smoking and its sub-variables.

Smoking habits among the respondents are also noteworthy, with nearly half of them, 45.36%, being smokers (Figure 5). The distribution of smoking duration is relatively even across different time spans, suggesting that smoking is a persistent habit among the respondents. The frequency of smoking leans towards daily use, with 59.09% smoking every day, and a significant number, 68.18%, smoking more than 10 sticks per day.

**Figure 6.**
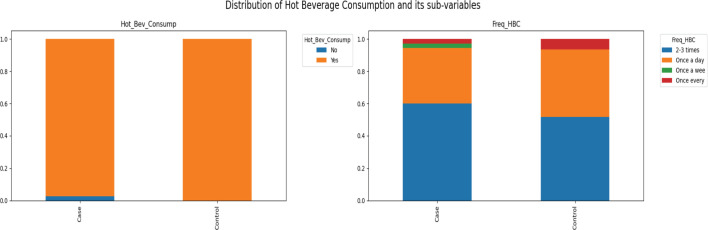
Distribution of hot beverage consumption and its sub-variables.

Hot beverage consumption is nearly universal among the respondents, with 98.97% reporting they consume hot beverages (Figure 6). The frequency of consumption is high, with 55.21% consuming hot beverages 2 and 3 times a day.

Table 2 describes the relationship between the duration of khat chewing and the risk of developing UDT cancers. The table provides the frequency and percentage of cases and controls for each duration category, along with the crude odds ratios (CORs) and adjusted odds ratios (AORs) with their 95% confidence intervals and corresponding *p*-values. No UDT cancer cases were reported among those chewing khat for less than 5 years. However, as the duration increased, so did the incidence of UDT cancer: 4.84% for 5–10 years, 11.29% for 10–20 years and 32.26% for over 20 years. Bivariate logistic regression indicates that individuals chewing khat for more than 20 years are 7.05 times more likely to develop UDT cancers compared to those chewing for less than 5 years (*p* < 0.05).

Table 3 describes the relationship between the frequency of khat chewing and the risk of developing UDT cancers. Higher frequencies of khat chewing correspond to increased incidence of UDT cancers: 33.87% among daily chewers, 11.29% among those chewing 4 days a week and 3.23% among those chewing twice a week. Notably, there are no UDT cancer cases among those chewing khat once a week. Statistical analysis shows a significant association, with frequent khat chewers having a 3.58 times higher chance of developing UDT cancers compared to less frequent chewers (*p* < 0.05). AOR confirms this association, indicating that frequent khat chewers are 6.89 times more likely to develop UDT cancers (*p* < 0.05).

Table 4 illustrates the relationship between the amount of khat chewed and the risk of developing UDT cancers. The highest consumption group (>600 g) has the most UDT cancer cases, at 37.10%. Statistical analysis reveals a COR of 3.15, indicating individuals in the highest consumption group are 3.15 times more likely to develop UDT cancers than those in the lowest consumption group (*p*-value = 0.003). The AOR of 6.60 after accounting for other factors confirms that high consumers are 6.60 times more likely to have UDT cancers (*p*-value = 0.030).

## Discussion

The study explored three primary aspects of khat chewing – duration, frequency and amount – and their association with UDT cancers. Long-term khat chewers (>20 years) showed a notable increase in UDT cancer incidence, with a significant COR of 7.05 (*p* < 0.001). However, the AOR was not statistically significant, suggesting potential confounding variables influencing this association. Frequent khat chewers, especially daily users, demonstrated a higher likelihood of UDT cancers, supported by a COR of 3.58 (*p* = 0.001) and an AOR of 6.89 (*p* = 0.032) after adjusting for other factors. Heavy khat consumption (>600 g) was significantly associated with increased UDT cancer cases, with a COR of 3.15 (*p* = 0.003) and an AOR of 6.60 (*p* = 0.030), indicating a dose-response relationship.

Our findings corroborate existing literature regarding the adverse health effects of khat chewing on UDT cancers. A study done in Ethiopia by Leon *et al* [11], found a two-fold elevation in EC risk in ever qat chewers compared with never users. Similar findings have been reported in a study done in Saudi Arabia by Alshahrani *et al* [4]. It was discovered that long-term khat users had nine somatic mutations in five cancer-related genes, compared to seven somatic mutations in four of the nine cancer-related genes carried by short-term users and it was concluded that Khat is a mutagenic and carcinogenic plant that provoked OSCC among short-term khat users (<15 years of use) and long-term users (>15 years of use).

The association between frequent khat chewing and increased UDT cancer incidence aligns with a study by Walle* et al* [16] concluded Frequent chewers were 7.58 times more likely to be affected by self-rated oral health problems compared to those who chewed less frequently (AOR: 7.58,95% CI:3.53–16.27). This finding also agrees with another study by Chong *et al* [7] which concluded that the prevalence of oral precancerous lesions increased significantly with increased frequency and khat caused premalignant oral lesions in dose- and time-dependent manner. Another study done in Yemen by Al-Jamaei *et al* [2] supported the association between khat and oral malignant, as well as potentially malignant oral disorders, highlighting that habitual khat chewing can induce oral erythroplakia, a premalignant lesion.

Moreover, our study’s findings on the dose-response relationship between khat consumption levels and UDT cancers are consistent with a study in Ethiopia by Walle *et al* [16] which found that chewers who consumed 100 g or more of khat in a single session had a 4.33-fold increased risk of oral premalignant lesions compared to those who chewed less. This is supported by another cross-sectional clinical sampling study by Kassie *et al* [10] (*n* = 109) and reported consumption of 100 g of khat per day significantly (*p* < 0.05) increased micronuclei and genetic damage in oral mucosa cells, indicating a dose-dependent rise in genotoxic effects and oral cancer risk among users.

The general study findings are consistent with a study which was done by Al-Maweri *et al* [3]in Yemen which reported that among 547 khat users, the presence of premalignant oral lesions was significantly associated (*p* < 0.001) with khat chewing only (without smoking). This also agrees with the retrospective study by Soufi *et al* [15] (*n* = 28) reported that 36% of the non-smoking, oro-pharyngeal cancer patients, had a history of khat chewing for at least 25 years. This study disagrees with a case-control study by Machoki *et al* [13] involving 91 cases and 182 controls, which found no significant association between khat usage and EC (*p* > 0.05).

## Strength and limitations

The study had several strengths. Controls were selected from cancer patients to ensure comparability and reduce selection bias, and participants were blinded to the study hypothesis to minimise recall bias. Key confounders, such as smoking, hot beverage consumption and sociodemographic factors, were controlled by using multivariate logistic regression, enhancing the internal validity of the study.

However, limitations included potential recall bias due to self-reported data, selection bias from recruiting participants at specific cancer clinics and a small sample size due to the limited availability of cancer clinics in Hargeisa, which may affect the generalisability of the results.

## Conclusion

The study investigated the relationship between khat chewing and UDT cancers among male patients in Hargeisa, Somaliland. The findings show an association between khat chewing and increased UDT cancer risk, highlighting duration, frequency and amount as key risk factors.

The study urges collaborative efforts among health organisations, government bodies and healthcare providers to develop targeted interventions and policies. These should include public awareness campaigns, cancer registry establishment, cessation programs and regulatory measures like quantity restrictions or taxation. Training for healthcare providers is crucial for early detection and management. Further research on khat’s long-term effects and carcinogenic properties is essential to inform evidence-based strategies and protect public health effectively.

## Proofreading assistance

ChatGPT was used to proofread this manuscript

## Conflicts of interest

The authors declare no conflicts of interest.

## Funding

This study did not receive any funding.

## Author contributions

AMA: Primary researcher, responsible for conducting the study, data collection, data analysis and writing the article.

MNM: Primary supervisor of the entire study, overseeing the research process and providing guidance.

AH: Assisted in study design and methodology and consulted on data analysis.

OMM: Assisted with data collection and contributed to the literature review.

## Figures and Tables

**Table 1.** Demographic and behavioral characteristics of the respondents.

Variable	Category	Frequency (*n* = 97)	Percentage (%)
1. Case or control	Control	61	62.89
	Case	36	37.11
2. Age group	18–34 years old	1	1.03
	35–54 years old	5	5.15
	55–64 years old	28	28.87
	>64 years old	63	64.95
3. Education level	No formal education	56	57.73
	Primary education	25	25.77
	Secondary education	13	13.40
	University education	3	3.09
4. Marital status	Single	15	15.46
	Married	78	80.41
	Divorced	3	3.09
	Widowed	1	1.03
5. UDT cancer	No	61	62.89
	Yes	36	37.11
6. Type of UDT cancer	Oral cancer	13	36.11
	EC	23	63.89
7. Khat chewing	No	35	36.01
	Yes	62	63.91
8. Duration of Khat chewing	<5 years	3	4.84
	5–10 years	16	25.81
	10–20 years	21	33.87
	>20 years	22	35.48
9. Frequency of Khat chewing	Once a week	3	4.84
	Twice a week	12	19.35
	4 days a week	22	35.48
	Every day	25	40.32
10. Amount of Khat chewing	100–300 g	12	19.35
	300–600 g	17	27.42
	>600 g	33	53.23
11. Smoking	No	53	54.64
	Yes	44	45.36
12. Duration of smoking	<5 years	5	11.36
	5–10 years	14	31.82
	10–20 years	11	25.00
	>20 years	14	31.82
13. Frequency of smoking	Twice a week	7	15.91
	4 days a week	11	25.00
	Every day	26	59.09
14 Amount of smoking	<5 sticks	8	18.18
	5–10 sticks	6	13.64
	>10 sticks	30	68.18
15. Hot beverage consumption	No	1	1.03
	Yes	96	98.97
16. Frequency of hot beverage consumption	Once a week	1	1.04
	Once every 2–4 days	5	5.21
	Once a day	37	38.54
	2–3 times a day	53	55.21
17. Clinic	Needle Hospital	42	43.30
	Nageeye Cancer Clinic	55	56.70

**Table 2.** Relationship between duration of Khat chewing and UDT cancers.

Variable	Category	Cases frequency (%)	Control frequency (%)	Total	COR (95%CI)	*p*-value	AOR (95% CI)	*p*-value
Duration of Khat chewing	<5 years	0 (0%)	3 (4.84%)	3 (4.84%)	7.051003	<0.0001	3.606747	0.182
	5–10 years	3 (4.84%)	13 (20.97%)	16 (25.81%)				
	10–20 years	7 (11.29%)	14 (22.58%)	21 (33.87%)				
	>20 years	20 (32.26%)	2 (3.23%)	22 (35.48%)				
Total		30 (48.39%)	32 (51.61%)	62 (100%)				

**Table 3.** Relationship between frequency of Khat chewing and UDT.

Variable	Category	Case frequency (%)	Control frequency (%)	Total	COR (95%CI)	*p*-value	AOR (95% CI)	*p*-value
Frequency of Khat chewing	Once a week	0 (0%)	3 (4.84%)	3 (4.84%)	3.580465	<0.01	6.892948	0.032
	Twice a week	2 (3.23%)	10 (16.13%)	12 (19.35%)				
	4 days a week	7 (11.29%)	15 (24.19%)	22 (35.48%)				
	Every day	21 (33.87%)	4 (6.45%)	25 (40.32%)				
Total		30 (48.39%)	32 (51.61%)	62 (100%)				

**Table 4.** Relationship between amount of Khat chewing and UDT.

Variable	Category	Case frequency (%)	Control frequency (%)	Total	COR (95%CI)	p-value	AOR (95% CI)	p-value
Amount of Khat chewing	100–300 g	1 (1.61%)	11 (17.74%)	12 (19.35%)	3.148575	<0.01	6.602866	0.030
	300–600 g	6 (9.68%)	11 (17.74%)	17 (27.42%)				
	>600 g	23 (37.10%)	10 (16.13%)	33 (53.23%)				
Total		30 (48.39%)	32 (51.61%)	62 (100%)				

**Table 5.** Bivariate logistic regression.

Status	Odds ratio	Std. Err.	*z*	*p*>|*z* |	[95% Conf. Interval]
Age group	1.447965	0.5117724	1.05	0.295	0.7242813 2.894735
Education level	0.4540243	0.1443598	2.48	0.013	0.2434638
Marital status	0.9426497	0.426756	0.13	0.896	0.3881456
Khat chewing	4.83871	2.493656	3.06	0.002	1.762212
Duration Khat chewing	7.051003	3.397833	4.05	0.000	2.741968
Freq of Khat chewing	3.580465	1.366335	3.34	0.001	1.694783
Amount of Khat Chewing	3.148575	1.195477	3.02	0.003	1.49597
Smoking	1.346154	0.5681727	0.70	0.481	0.5886092
Duration of smoking	1.034315	0.3218092	0.11	0.914	0.5621036
Freq of smoking	2.793771	1.42271	2.02	0.044	1.029727
Amount of smoking	1.375205	0.5636939	0.78	0.437	0.6158308
Freq HBC	1.144305	0.3822426	0.40	0.687	0.5945754

**Table 6.** Multivariate logistic regression.

Status	Odds ratio	Std. Err.	*z*	*p*>|*z* |	[95% Conf. Interval]
Education level	1.403736	2.045511	0.23	0.816	0.0807091
Khat chewing	4.83871	2.493656	3.06	0.002	1.762212
Duration of Khat chewing	3.606747	3.467148	1.33	0.182	0.5481002
Freq of Khat chewing	6.892948	6.200958	2.15	0.032	1.182106
Amount of Khat of chewing	6.602866	5.725905	2.18	0.030	1.206656
Freq of smoking	6.52864	6.662971	1.84	0.066	0.8832976

**Table 7.** Socio-demographic characteristics and behavioral characteristics of cases and controls.

Variable	Case frequency (%)	Control frequency (%)	Total
Age group			
• 18–34 years	0 (0%)	1 (1.03%)	1 (1.03%)
• 35–54 years	2 (2.06%)	3 (3.09%)	5 (5.15%)
• 55–64 years	8 (8.25%)	20 (20.62%)	28 (28.87%)
• >64 years	26 (26.80%)	37 (38.14%)	63 (64.95%)
Education level			
• No formal	28 (28.87%)	28 (28.87%)	56 (57.73%)
• Primary ed.	5 (5.15%)	20 (20.62%)	25 (25.77%)
• Secondary	2 (2.06%)	11 (11.34%)	13 (13.40%)
• University	1 (1.03%)	2 (2.06%)	3 (3.09%)
Marital status			
• Single	4 (4.12%)	11 (11.34%)	15 (15.46%)
• Married	32 (32.99%)	46 (47.42%)	78 (80.41%)
• Divorced	0 (0%)	3 (3.09%)	3 (3.09%)
• Widowed	0 (0%)	1 (1.03%)	1 (1.03%)
• UDT cancer			
• No	0 (0%)	61 (62.89%)	61 (62.89%)
• Yes	36 (37.11%)	0 (0%)	36 (37.11%)
Type of UDT cancer			
• Oral cancer	13 (35.14%)	-	13 (35.14%)
• Esophageal	23 (64.86%)	-	23 (64.86%)
Khat chewing			
• No	6 (6.19%)	30 (30.93%)	36 (37.11%)
• Yes	30 (30.93%)	31 (31.96%)	61 (62.89%)
Duration of Khat chewing			
• <5 years	0 (0%)	3 (4.84%)	3 (4.84%)
• 5–10 years	3 (4.84%)	13 (20.97%)	16 (25.81%)
• 10–20 years	7 (11.29%)	14 (22.58%)	21 (33.87%)
• >20 years	20 (32.26%)	2 (3.23%)	22 (35.48%)
Frequency of Khat chewing			
• Once a week	0 (0%)	3 (4.84%)	3 (4.84%)
• Twice a week	2 (3.23%)	10 (16.13%)	12 (19.35%)
• 4 days a week	7 (11.29%)	15 (24.19%)	22 (35.48%)
• Every day	21 (33.87%)	4 (6.45%)	25 (40.32%)
Amount of Khat chewing			
• 100–300 g	1 (1.61%)	11 (17.74%)	12 (19.35%)
• 300–600 g	6 (9.68%)	11 (17.74%)	17 (27.42%)
• >600 g	23 (37.10%)	10 (16.13%)	33 (53.23%)
Smoking			
• No	18 (18.56%)	35 (36.08%)	53 (54.64%)
• Yes	18 (18.56%)	26 (26.80%)	44 (45.36%)
Duration of smoking			
• <5 years	4 (9.09%)	1 (2.27%)	5 (11.36%)
• 5–10 years	3 (6.82%)	11 (25.00%)	14 (31.82%)
• 10–20 years	3 (6.82%)	8 (18.18%)	11 (25.00%)
• >20 years	8 (18.18%)	6 (13.64%)	14 (31.82%)
Frequency of smoking			
• Twice a week	1 (2.27%)	6 (13.64%)	7 (15.91%)
• 4 days a week	3 (6.82%)	8 (18.18%)	11 (25.00%)
• Every day	14 (31.82%)	12 (27.27%)	26 (59.09%)
Amount of smoking			
• <5 sticks	2 (4.55%)	6 (13.64%)	8 (18.18%)
• 5–10 sticks	3 (6.82%)	3 (6.82%)	6 (13.64%)
• >10 sticks	13 (29.55%)	17 (38.64%)	30 (68.18%)
Hot beverage consumption			
• No	1 (1.03%)	0 (0%)	1 (1.03%)
• Yes	35 (36.08%)	61 (62.89%)	96 (98.97%)
Frequency of hot beverage consumption			
• Once a week	1 (1.04%)	0 (0%)	1 (1.04%)
• Once every	1 (1.04%)	4 (4.17%)	5 (5.21%)
• Once a day	12 (12.50%)	25 (26.04%)	37 (38.54%)
• 2–3 times	21 (21.88%)	32 (33.33%)	53 (55.21%)
